# AP39, a Mitochondrial-Targeted H_2_S Donor, Improves Porcine Islet Survival in Culture

**DOI:** 10.3390/jcm11185385

**Published:** 2022-09-14

**Authors:** Misaki Shinzato, Chika Miyagi-Shiohira, Kazuho Kuwae, Kai Nishime, Yoshihito Tamaki, Tasuku Yonaha, Mayuko Sakai-Yonaha, Ikuo Yamasaki, Ryusei Otsuka, Issei Saitoh, Masami Watanabe, Hirofumi Noguchi

**Affiliations:** 1 Department of Regenerative Medicine, Graduate School of Medicine, University of the Ryukyus, Okinawa 903-0215, Japan; 2 Department of Pediatric Dentistry, Asahi University School of Dentistry, Hozumi 501-0296, Japan; 3 Department of Urology, Okayama University Graduate School of Medicine, Dentistry and Pharmaceutical Sciences, Okayama 700-8558, Japan

**Keywords:** AP39, hydrogen sulfide donor (H_2_S), reactive oxygen species (ROS), islet transplantation, islet culture

## Abstract

The rapid deterioration of transplanted islets in culture is a well-established phenomenon. We recently reported that pancreas preservation with AP39 reduces reactive oxygen species (ROS) production and improves islet graft function. In this study, we investigated whether the addition of AP39 to the culture medium could reduce isolated islet deterioration and improve islet function. Isolated islets from porcine pancreata were cultured with 400 nM AP39 or without AP39 at 37 °C. After culturing for 6–72 h, the islet equivalents of porcine islets in the AP39(+) group were significantly higher than those in the AP39(−) group. The islets in the AP39(+) group exhibited significantly decreased levels of ROS production compared to the islets in the AP39(−) group. The islets in the AP39(+) group exhibited significantly increased mitochondrial membrane potential compared to the islets in the AP39(−) group. A marginal number (1500 IEs) of cultured islets from each group was then transplanted into streptozotocin-induced diabetic mice. Culturing isolated islets with AP39 improved islet transplantation outcomes in streptozotocin-induced diabetic mice. The addition of AP39 in culture medium reduces islet deterioration and furthers the advancements in β-cell replacement therapy.

## 1. Introduction

Pancreatic islet transplantation offers a minimally invasive option for treatment of type 1 diabetes [1,2,3,4,5]. Culturing isolated islets before transplantation is beneficial as it reduces exocrine contamination of transplanted tissue. Moreover, it allows time for additional quality control testing and induction of immunosuppressive protocols, and is advantageous for recipients living far away from transplant institutes [3,4,6,7]. The culture of isolated islets may reduce their immunogenicity by depleting viable hematogenous and lymphoid cells [8]. Therefore, many centers culture isolated islets before clinical transplantation [3,4,6,7]. However, scientific evidence indicates that isolated islets deteriorate rapidly in culture [6,9,10,11] and islet reduction during culture results in lower transplantation rates.

Hydrogen sulfide (H_2_S) has emerged as an important signaling molecule in vivo and contributes to several physiological and pathological processes [12]. H_2_S has been shown to exhibit anti-apoptotic, anti-inflammatory, and antioxidant effects that protect the kidneys from warm ischemic injury [13,14,15] and the heart against ischemia-reperfusion injury (IRI) [16,17,18]. AP39 [(10-oxo-10-(4-(3-thioxo-3H-1,2-dithiol-5yl) phenoxy)decyl) triphenylphosphonium bromide] is a representative of a class of donors known as mitochondrial-targeted H_2_S, and is a synthetic H_2_S donor that consists of a mitochondria-targeting triphenylphosphonium motif [19]. AP39 delivers H_2_S into the mitochondria, where it attenuates mitochondrial reactive oxygen species (ROS) generation and preserves mitochondrial integrity. Moreover, AP39 can protect cells against IRI in the kidneys, brain, and myocardia [20,21,22]. We recently reported that pancreas preservation with AP39 reduces ROS production and improves islet graft function [23].

In this study, we investigated whether the addition of AP39 to the culture medium could reduce the deterioration of isolated islets and improve islet function. Porcine pancreata were used for experimentation. In Japan, it is illegal to divert organs harvested from cadavers for human organ transplantation for research purposes. Therefore, it is difficult to obtain organs for research even when the organs are for human tissue transplantation, despite the lack of laws regulating the use of harvested tissues from cadaveric donors. Moreover, porcine islets are expected to be used for islet xenotransplantation [24,25,26,27,28,29].

## 2. Materials and Methods

### 2.1. Procurement, Preservation, and Islet Isolation of Porcine Pancreas

Procurement, preservation, and islet isolation of porcine pancreas were performed as previously described [5,29,30]. Briefly, porcine pancreata (three years old; female animal) were obtained from a local slaughterhouse. The local slaughterhouse removed the animal’s blood and separated the porcine body and abdominal organs including the pancreas (approximately 10 min), and we started the procurement (approximately 7 min). A catheter was inserted into the main pancreatic duct and 1 mL/g of pancreas weight of modified extracellular-type trehalose-containing Kyoto (MK) solution (ETK solution (Otsuka Pharmaceutical Factory, Naruto, Japan) with ulinastatin), was infused through the intraductal cannula (approximately 11 min) [30]. The pancreata were stored in MK solution at 4 °C for approximately 18 h. Operation time was defined as the time elapsed between the start of the operation and the removal of the pancreas. Warm ischemic time (WIT) was defined as the time elapsed between the removal of the animal’s blood and placement of the pancreas into the preservation solution (approximately 10 + 7 + 11 = 28 min). Cold ischemic time (CIT) was defined as the time elapsed between the placement of the pancreas into the preservation solution and the start of islet isolation. Porcine islet isolation was performed as previously described [1,2,23,31,32,33,34].

### 2.2. Culture of Isolated Porcine Islets

Isolated porcine islets were divided and incubated with Connaught Medical Research Laboratories (CMRL) 1066 (Sigma-Aldrich Japan, Tokyo, Japan) medium supplemented with insulin (10 mg/L), transferrin (5.5 mg/mL), selenium (0.0067 mg/mL) solution (ITS-G solution; Thermo Fisher Scientific, Tokyo, Japan), and 0.5% human serum albumin (Sigma-Aldrich, Tokyo, Japan) for 6, 24, 48, and 72 h with 400 nM AP39 or without AP39 in an incubator set at 37 °C under 5% CO_2_ and 95% air. The concentration of AP39 was determined based on our previous data [23].

### 2.3. Islet Evaluation

Dithizone (DTZ; 3 mg/mL, final concentration: Sigma-Aldrich, Tokyo, Japan) staining and double fluorescein diacetate/propidium iodide (FDA/PI; Sigma-Aldrich, Tokyo, Japan) staining were performed as previously described [29,35,36,37]. The rudimentary number of islets in each diameter class was determined by counting the islets after DTZ staining using an optical graticule. This number was then converted to the standard number of islet equivalents (IEs; diameter standardized to 150 μm) [35]. Both evaluations were performed in a blinded manner by two evaluators.

Gross morphology (score) was qualitatively assessed by two independent investigators scoring the islets for shape (flat vs. spherical), border (irregular vs. well-rounded), integrity (fragmented vs. solid or compact), uniformity of staining (not uniform vs. perfectly uniform), and diameter (least desirable: all cells < 100 μm; most desirable: more than 10% of the cells >200 μm) [29]. Each parameter was graded from zero to two with zero being the worst and two the best score, so that the worst islet preparations were given a cumulative score of zero and the best a score of ten. Spherical, well-rounded, solid or compact, uniformly stained, and large islets were characterized as the best [29].

### 2.4. Annexin V/PI Staining

Islet viability was assessed after separation of islet cells using FACSAria (*n* = 6 each, from 6 islet isolations). A total of 1000 IEs per group were separated using Accutase (Innovative Cell Technologies), and fluorescent annexin V/PI staining was performed as previously described [29]. Briefly, single cells were incubated for 15 min at room temperature in 100 μL of Annexin V Incubation Reagent (10 μL of 10× Binding Buffer, 10 μL of PI, 1 μL of TACS^TM^ Annexin V-FITC, and 79 μL of distilled water; TACS^TM^ Annexin V-FITC kit; Trevigen, Gaithersburg, MD, USA). A total of 10,000 cells were analyzed using FACSAria (Becton Dickinson, Franklin Lakes, NJ, USA). Cells were not gated for live cells because we evaluated apoptotic and necrotic cells together with live cells in this experiment.

### 2.5. In Vitro Assessment

ROS production and mitochondrial membrane permeability were evaluated as previously described [23]. Briefly, cellular ROS production was assessed by staining the dissociated islet cells with 10 μM dihydrorhodamine-123 (DHR-123; Thermo Fisher Scientific K.K., Tokyo, Japan). We dispersed 1000 IEs of the cultured islets (24 h) in each group into single cells following treatment with Accutase (Innovative Cell Technologies, La Jolla, CA, USA) at 37 °C for 15 min. After two washes with phosphate-buffered saline (PBS) supplemented with 3% fetal bovine serum (FBS), single cells were incubated in 10 μM DHR-123 for 15 min at room temperature.

Mitochondrial membrane permeability and proton pumping was assessed by staining the dissociated islet cells with 5 μM JC-1 dye (Thermo Fisher Scientific K.K., Tokyo, Japan). We dispersed 1000 IEs of cultured islets (24 h) in each group into single cells following treatment with Accutase at 37 °C for 15 min. After two washes with PBS supplemented with 3% FBS, the single cells were incubated with 5 μM JC-1 dye for 15 min at room temperature.

To measure the production of adenosine triphosphate (ATP), the cultured islets (24 h; 10 IEs) in each group were washed twice with PBS at 4 °C and solubilized. The amount of ATP was measured using an ATP assay system (Toyo Ink, Tokyo, Japan), according to the manufacturer’s instructions. Briefly, after incubating at room temperature, 100 μL reagent was added to 10 μL of the cell extract. The samples were then measured using a luminometer.

Islet function was assessed by monitoring insulin secretion from the purified islets following glucose stimulation, according to the procedure described by Shapiro et al. [1]. Briefly, 1200 IEs of the cultured islets (24 h) in each group were preincubated with Roswell Park Memorial Institute 1640 (RPMI 1640; Sigma-Aldrich, Tokyo, Japan) medium, containing 2.8 mM glucose for 1 h in an incubator set at 37 °C and 5% CO_2_. The islets were then incubated with either 2.8 mM or 25 mM of glucose in RPMI 1640 for 2 h. The supernatant was collected, and insulin levels were determined using a commercially available enzyme-linked immunosorbent assay (ELISA) kit (ALPCO Insulin ELISA kit; ALPO Diagnostics, Windham, NH, USA). The stimulation index was calculated as the ratio of insulin released from the islets during stimulation with a high glucose concentration to insulin released during stimulation with a low glucose concentration. The islets were then sonicated in distilled water to determine intracellular insulin and intracellular total protein content, as previously described [38].

### 2.6. In Vivo Assessment

Isolated islets were incubated with 400 nM AP39 or without AP39 for 6 h. A total of 1500 IEs of cultured islets were processed for transplantation. In a preliminary study, approximately 70% of mice were normoglycemic after transplantation of 2000 IEs of 6 h cultured islets (without AP39). Approximately 40% were normoglycemic after transplantation of 1750 IEs, and less than 5% were normoglycemic after transplantation of 1500 IEs. Therefore, 1500 IEs of 6 h cultured islets were processed for transplantation into each group. Diabetes induction by streptozotocin, and transplantation into diabetic or severe combined immunodeficient (SCID) mice (six weeks old; male mice: Charles River Laboratories Japan, Inc., Kanagawa, Japan) were performed as previously described [25,28,35]. Hyperglycemia was defined as a glucose level of >350 mg/dL detected twice consecutively after STZ injection. Normoglycemia was defined when two consecutive blood glucose level measurements showed less than 200 mg/dL. The kidney implanted porcine islets were removed 30 days post-transplantation. All animal studies were approved by the Institutional Animal Care and Use Committee of the University of the Ryukyus.

### 2.7. Statistical Analysis

All data are expressed as mean ± standard error (SE). Microsoft Excel was used to perform Student’s *t*-test to compare two samples from independent groups. The differences in the duration of graft survival between the groups were evaluated using the Kaplan–Meier log-rank test and StatView software. Statistical significance was set at *p* < 0.05.

## 3. Results

### 3.1. Culture of Isolated Islets with AP39

**Figure 1:**
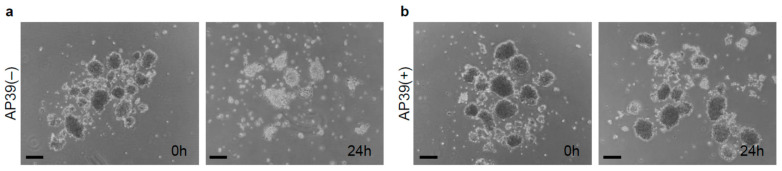
Culture of isolated islets with AP39. (**a**) Culture of isolated islets without AP39. Porcine islets were incubated without AP39 at 37 °C for 24 h. (**b**) Culture of isolated islets with AP39. Porcine islets were incubated with 400 nM AP39 at 37 °C for 24 h. Scale bars = 200 μm.

**Figure 2:**
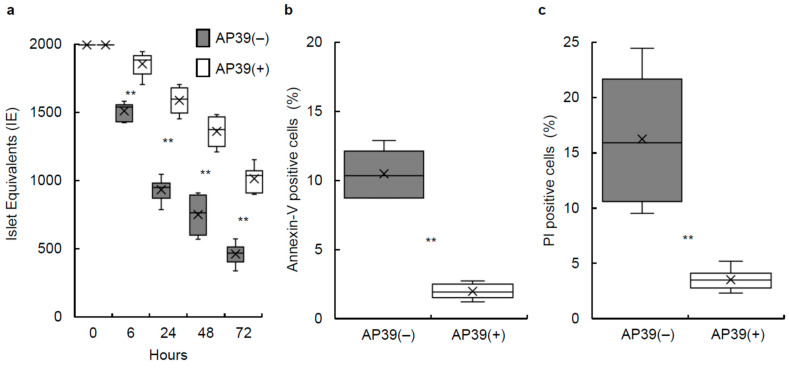
Effect of AP39 on cultured porcine islets. (**a**) Number of porcine islets after culture. 2000 IEs of porcine islets were cultured with 400 nM AP39 or without AP39 for 72 h. After 6, 24, 48, and 72 h of culturing with or without AP39, the islets were counted to calculate the IE (*n* = 6 each). (**b**,**c**) Annexin V/propidium iodide (FDA/PI) staining of 24 h cultured islets. We dispersed 1000 total IEs of cultured islets with or without AP39 into single cells using Accutase and then incubated the single cells with FITC-annexin-V (**b**) and PI (**c**). AP39(−) group: *n* = 6; AP39(+) group: *n* = 6. Data are expressed as the mean ± SE. ** *p* < 0.01.

We evaluated the effects of AP39 on pancreatic islets in culture. Porcine islets were isolated (*n* = 6) and pancreas tissues and isolated islets were characterized to make sure that adequate quality islets were used in this study, as shown in Table 1 and Table 2. Porcine islets were then cultured with 400 nM AP39 or without AP39 at 37 °C for 24 h. AP39(−) porcine islets displayed greater reduction in size (islet equivalents) compared to the AP39(+) group (Figure 1). To investigate whether AP39 could prevent islet reduction during culturing, the IE after culturing for 6, 24, 48, and 72 h was compared between the two groups. The IE of islets cultured with 400 nM AP39 (*n* = 6) was significantly higher than that without AP39 (*n* = 6) (Figure 2a), suggesting that AP39 prevented the decline in the number of porcine islets during culture.

### 3.2. Annexin V/PI Staining

To examine whether AP39 prevents islet apoptosis and/or necrosis, Annexin V and PI assays were performed. Annexin V staining detects early apoptosis whereas PI staining can be used to identify late apoptotic or necrotic cells. Approximately 1000 IEs of 24 h cultured islets, with 400 nM AP39 or without AP39, were dispersed using Accutase. Cells were then treated with annexin V and PI. The percentages of annexin V-positive cells in the AP39(−) and AP39(+) groups (*n* = 6 each, from 6 islet isolations) were 10.4 ± 0.7% and 2.1 ± 0.2%, respectively (Figure 2b). The percentages of PI-positive cells in the AP39(−) and AP39(+) groups were 15.8 ± 2.4% and 3.4 ± 0.4%, respectively (Figure 2c). The apoptosis and necrosis rates were significantly lower in the AP39(+) group than in the AP39(−) group.

### 3.3. Production of ROS in Porcine Islets

**Figure 3:**
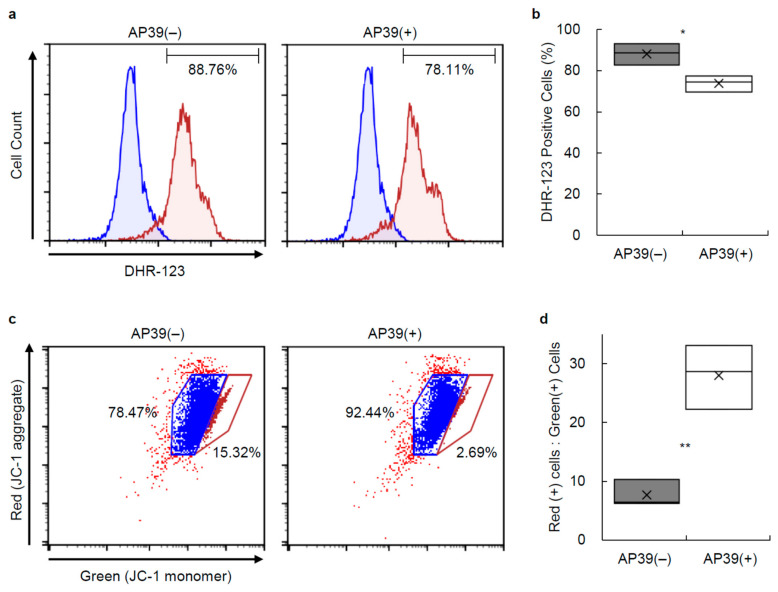
Production level of ROS in cultured islet cells and JC-1 staining of cultured islet cells. (**a**,**b**) ROS production in 24 h cultured islets with or without AP39 was measured via flow cytometry using staining of dihydrorhodamine-123 (DHR-123). Control cells were not stained with DHR-123. (**a**) Representative plots of DHR-123 fluorescence in dispersed cells. (**b**) Percentage of DHR-123 positive cells. (**c**,**d**) Mitochondrial membrane potential of 24 h cultured islets with or without AP39 was measured via flow cytometry using JC-1 staining. (**c**) Representative plots of JC-1 staining of dispersed cells. (**d**) Ratio of red (+) to green (+) cells in AP39(+) and AP39(−) groups. Higher values indicate more cells with polarized (healthy) mitochondria. AP39(+) and AP39(−) groups: *n* = 3 each. Data are expressed as the mean ± SE. * *p* < 0.05. ** *p* < 0.01.

To evaluate the production of ROS in porcine islets, 1000 IEs of 24 h cultured islets treated with 400 nM AP39 (*n* = 3, from 3 isolations) or without AP39 (*n* = 3 from the same three isolations) were dispersed into single cells and stained with 10 μM DHR-123. The porcine islets cultured with AP39 exhibited significantly decreased production levels of ROS compared to those cultured without AP39 (Figure 3a,b).

### 3.4. Mitochondrial Membrane Permeability and Proton Pumping in Porcine Islets

To evaluate mitochondrial membrane permeability and proton pumping in porcine islets, 1000 IEs of islets cultured for 24 h with 400 nM AP39 (*n* = 3, from 3 isolations) or without AP39 (*n* = 3 from the same three isolations) were dispersed into single cells and stained with 5 μM JC-1 dye. The cultured AP39(+) islets exhibited significantly increased mitochondrial membrane and proton pumping potential compared to those cultured without AP39 (Figure 3c,d).

### 3.5. Stimulation Index of Cultured Islets Treated with or without AP39

**Figure 4:**
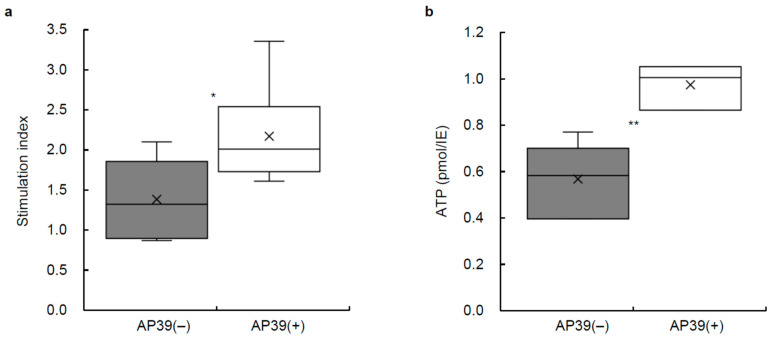
Stimulation index and ATP content of cultured islets with or without AP39. (**a**) The stimulation index was measured after 24 h islet culture in each group. AP39(+) and AP39(−) groups: *n* = 6 each, * *p* < 0.05. (**b**) ATP content of cell lysates after 24 h islet culture was assessed using an ATP assay system. ATP content was normalized to DNA content. AP39(+) and AP39(−) groups: *n* = 6 each, ** *p* < 0.01. Data are expressed as the mean ± SE.

The stimulation index of the 24 h cultured islets was measured to assess islet function in vitro. The stimulation index values for the AP39(+) group (*n* = 6 from six isolations) were significantly higher than those in the AP39(−) group (*n* = 6 from the same six isolations) with 2.05 ± 0.22 and 1.32 ± 0.19, respectively (*p* < 0.05; Figure 4a).

### 3.6. ATP Content of Cultured Islets Treated with or without AP39

The ATP content of the cell lysate after 24 h of culture with 400 nM AP39 or without AP39 was measured using an ATP assay system. The ATP content was significantly higher in porcine islets cultured with 400 nM AP39 (*n* = 6; 1.02 ± 0.04 pmol/IE) compared to cultured islets without AP39 (*n* = 6; 0.58 ± 0.07 pmol/IE) (*p* < 0.01; Figure 4b).

### 3.7. In Vivo Assessment of Cultured Islets Treated with or without AP39

To evaluate the graft function of porcine islets in vivo, marginal numbered islets (1500 IEs) cultured with 400 nM AP39 or without AP39 (*n* = 12 from 6 islet isolations, *n* = 2 per isolation) were implanted below the kidney capsule of diabetic SCID mice. We used 6 h cultured islets because the results shown in Figure 2a confirmed a significant difference at 6 h. None of the 12 mice implanted with porcine islets cultured without AP39 became normoglycemic (Table 3). In contrast, the blood glucose levels of 7 out of 12 mice (58.3%) implanted with porcine islets cultured with 400 nM AP39 gradually decreased and reached the normoglycemic range (Table 3). The number of mice that became normoglycemic post-transplantation between the AP39(+) group and AP39(−) group were significantly different *(p* < 0.01). These results reveal that the addition of AP39 to the culture medium improved islet graft function.

## 4. Discussion

In this study, we showed that the addition of AP39 to the culture medium reduced the deterioration of isolated islets and improved function of porcine islets. Multiple H_2_S donors are available and have been extensively investigated in diverse pathological processes including IRI in different models of solid organ transplantation (heart, lung, kidney, liver, intestine, and pancreas) [12,39,40]. GYY4137 is widely used as a research tool to study the effects of H_2_S. Lobb et al. compared H_2_S donors AP39 and GYY4137 in vitro using rat kidney epithelial cells (NRK-52) during cold hypoxic injury [41]. Treatment with AP39 improved the protective capacity of H_2_S > 1000-fold compared to similar levels of the GYY4137. Other H_2_S donors, ATB-346 and SG1002, have completed Phase I clinical trials in healthy volunteers, and GIC-1001 has been shown to be safe in a Phase I clinical trial as well as now in Phase II clinical trials as a pre-colonoscopy analgesic [12].

We previously reported that pancreas preservation with AP39 reduces ROS production and improves islet graft function [23]. The apparent effects of AP39 when added to pancreas preservation solutions, or when added to media used for islet culture are similar. H_2_S has protective effects against IRI in several organs [22,41,42], and the mitochondria in these cells are the primary site of H_2_S activity [17,43,44]. It has been reported that the cytosolic H_2_S producing enzyme cystathionine-gammalyase can translocate to the mitochondria during cell damage, presumably to produce H_2_S, where it mediates responses against cell damage [45]. AP39 is a synthetic donor that supplies H_2_S in a controlled manner similar to physiological production in the latter. AP39 contains a cationic triphenylphosphonium group that allows the compound to home into the mitochondria before supplying H_2_S [19,46]. The effect of AP39 has been reported in the brain [20], kidneys [21,39,42], and heart [22]. In a renal IRI model, AP39 protects renal injury, oxidative stress, and inflammation when reperfused in vivo [21]. AP39 significantly limits infarct size and recovers post-ischemic function [22]. Our study suggested that AP39 reduced ROS production and preserved mitochondrial membrane integrity in isolated islets in culture, as compared to isolated islets cultured without AP39. This suggests that treatment with AP39 in culture improves cellular viability by reducing ROS production and preserving mitochondrial membrane integrity.

We measured ATP content in cultured islets. Low levels of H_2_S have been shown to stimulate ATP production through the donation of electrons to the electron transport chain [47,48] and the improvement of mitochondrial integrity (Figure 3). ATP content was significantly higher in the AP39(+) group than in the AP39(−) group. Thus, AP39 can induce ATP production in isolated islets during culture.

One mechanism reducing islet mass during culture is cell death by apoptosis and activation of intracellular death signaling pathways [49,50,51,52]. Our results showed that culturing isolated islets with AP39 before transplantation can prevent islet apoptosis during culture and improve outcomes in pancreatic islet transplantation. It has also been reported that oxygen depletion in islet cores results in central necrosis during culture [52,53]. Central necrosis is another reason for deterioration during islet culture. Our data in Figure 2c show that the rate of PI staining, which can be used to identify late apoptotic or necrotic cells, was significantly lower in the AP39(+) group than in the AP39(−) group, suggesting that islet culture with AP39 may also prevent islet necrosis. To date, no report describes a relationship between islet isolation or culture and pyroptosis. We evaluated caspase 1, a key initiator of the canonical pyroptosis pathway, in cultured islets and found that it was not activated.

## 5. Conclusions

Treatment of porcine islets with AP39 in culture improved islet graft function. Although many institutes have introduced the culturing of human islets prior to transplantation [3,4,6,7], it is well-documented that isolated islets deteriorate rapidly in culture [6,9,10,11]. Our results further the advances in β-cell replacement therapy by proposing to culture isolated islets with AP39 before transplantation as a potentially viable treatment option for individuals afflicted with type 1 diabetes.

## Figures and Tables

**Table 1:** The characteristics of the tissue and procedures.

Characteristic	*n* = 6
Pancreas size (g)	105.8 ± 7.0
Operation time (min)	7.2 ± 1.1
Warm ischemic time (min)	28.2 ± 1.1
Cold ischemic time (min)	1114.2 ± 13.9
Phase I period (min)	11.0 ± 0.4
Phase II period (min)	38.8 ± 4.0
Undigested tissue (g)	8.8 ± 1.7

The data are expressed as the mean ± SE.

**Table 2:** The islet characteristics.

Characteristic	*n* = 6
IE before purification	544,440 ± 89,511
IE after purification	458,933 ± 96,123
Embedded islets (%)	11.5 ± 3.3
Viability (%)	96.6 ± 0.4
Purity (%)	57.3 ± 4.4
Post-purification recovery (%) ^1^	82.2 ± 5.5
Score	9.4 ± 0.1

The data are expressed as the mean ± SE. ^1^ Post-purification recovery (%) = IE after purification/IE before purification × 100.

**Table 3:** In vivo functional assay.

	**AP39(−)**	**AP39(+)**
No. Transplanted Mice	12	12
No. Cured Mice	0	7
%	0	58.3
*p*-Value vs. AP39(−)		<0.01

Marginal numbered porcine islets (1500 IEs) cultured with or without AP39 were transplanted into immunodeficient diabetic mice. The normoglycemia was defined as <200 mg/dL in non-fasting condition in more than 2 consecutive days.

## Data Availability

The data that support the findings of this study are available from the corresponding author, H.N., upon reasonable request.
